# Assessing efficacy and safety of replacement fluids in therapeutic plasma exchange: A systematic scoping review of outcome measures used

**DOI:** 10.1002/jca.21996

**Published:** 2022-06-21

**Authors:** Ruchika Kohli, Louise J. Geneen, Susan J. Brunskill, Carolyn Doree, Lise Estcourt, Stephan E. J. Chee, Raya Al‐Bader, Wing Y. C. Sin, Peter MacCallum, Laura Green

**Affiliations:** ^1^ Wolfson Institute of Population Health, Queen Mary University of London London; ^2^ Systematic Review Initiative, National Health Service Blood and Transplant Oxford UK; ^3^ Nuffield Division of Clinical Laboratory Sciences, Radcliffe Department of Medicine University of Oxford Oxford UK; ^4^ Barts and the London, School of Medicine and Dentistry London UK; ^5^ East London NHS Foundation Trust London UK; ^6^ Barts Health NHS Trust London UK; ^7^ National Health Service Blood and Transplant London UK; ^8^ Blizard Institute, Queen Mary University of London London UK

**Keywords:** immunology, neurology, plasma exchange

## Abstract

**Objective:**

The aim of this systematic scoping review is to identify and categorize the outcome measures that have been reported in clinical studies, where therapeutic plasma exchange (TPE) has been used as an intervention in any clinical settings, excluding thrombotic thrombocytopenic purpura (TTP).

**Methods:**

We searched electronic databases using a predefined search strategy from inception to October 9, 2020. Two reviewers independently screened and extracted data.

**Results:**

We included 42 studies (37 RCTs and 5 prospective cohort studies) grouped into six main categories (neurology, immunology, renal, rheumatology, hematology, and dermatology). Primary outcomes were defined in eight studies (19%, 8/42) and were categorized as efficacy (five studies) or patient reported outcomes (three studies). A power calculation was reported in six studies (75%, 6/8): five neurology studies (mainly patient reported outcomes) and a single immunological study (efficacy outcome). Disease‐specific efficacy outcomes were dependent on the clinical setting of the population receiving TPE. Most of the trials (43%, 18/42) were undertaken in patients with neurology conditions where clear, disease‐specific, clinical outcome measures were used, including neurological disability scales (11/18, 61%), change in neurological examination (9/18, 50%), and functional improvement scores (7/18, 39%). For other conditions, the reporting of disease‐specific outcomes was poorly reported. Safety outcomes were mainly related to replacement fluid type rather than being disease‐specific. The most common outcome reported was hypotension (19%, 8/42), and this was primarily in patients exchanged with albumin.

**Conclusion:**

Future clinical studies to determine which fluid replacement option is most efficacious and safe should use disease‐specific outcomes, as a trial in one therapeutic area may not necessarily translate to another therapeutic area. Patient reported outcomes are not universally reported for all disease areas. Safety measures focused primarily on fluid safety.

## 1 INTRODUCTION

Therapeutic plasma exchange (TPE) is a process involving the extracorporeal separation of plasma from the cellular components of blood and exchanging it with replacement physiologic fluids.^1^, ^2^, ^3^ Examples of physiological fluids used for replacement during TPE include fresh frozen plasma (FFP), 5% human albumin solution (5% HAS), colloids (eg, Gelofusine) and crystalloids (0.9% normal saline).^4^ The type and severity of the disease being treated dictate the intensity and duration of TPE treatments.^5^

The American Society for Apheresis (ASFA) Guidelines on the Use of Therapeutic Apheresis in Clinical Practice has published the recommendations^6^ on the type of fluid that should be used for different conditions, with grading of evidence for most clinical indications being given as low (ie, grade 2B‐2C), indicating that the evidence for the efficacy and safety of different fluids is lacking. The most common clinical specialties where TPE is provided are neurological (eg, acute Guillain‐Barré syndrome, chronic inflammatory demyelinating polyneuropathy, myasthenia gravis etc.), renal (eg, Goodpasture's syndrome, antineutrophil cytoplasmic antibody‐associated rapidly progressive glomerulonephritis), and hematological diseases (eg, thrombotic thrombocytopenic purpura (TTP), thrombotic microangiopathy, etc.).

The main factors for choosing a replacement fluid during TPE are dependent on availability of the fluid type and its cost, patient's baseline bleeding risk, and the underlying disease condition.^7^ For example, FFP is the preferred fluid for TPE in patients who are at risk of bleeding, who have thrombotic TTP, or who are due to undergo major surgeries where bleeding risk could be high (eg, renal transplantation).^8^, ^9^ In TTP, replacement fluid with plasma provides a source of functional A Disintegrin and Metalloprotease with ThromboSpondin type 1 motif, member 13 (ADAMTS13) that is lacking in these patients, and following TPE treatment with plasma, clinical outcome measures that are disease specific for TTP are already used to monitor the effect of TPE (eg, mortality, measurement of ADAMTS13 levels, platelet count, and lactate dehydrogenase level).^9^ Such clarity in monitoring the efficacy and safety of other fluid replacement therapies (eg, HAS, colloids, or crystalloids) does not exist. To improve the evidence of appropriate fluid therapies for TPE, we need to first understand the outcome measures that are relevant for evaluating the efficacy, safety, and cost‐effectiveness of fluids in different clinical conditions, as has been developed for TTP.

The aim of this systematic scoping review is to identify and categorize the outcome measures that have been reported in clinical studies where TPE has been used as an intervention in any clinical settings, excluding TTP. These data will help to inform future trials that aim at comparing the efficacy and safety of different replacement fluid used for TPE.

## 2 METHODS

This review was conducted in accordance with published guidelines for conducting scoping reviews^10^ and is reported as per the Preferred Reporting Items for Systematic Reviews and Meta‐analysis (PRISMA) guidelines.^11^ The review was prospectively developed and registered on Open Science Framework (registration number 10.17605/OSF.IO/3HDA7). Post registration, the protocol was amended to: (1) expand the scope of clinical indications for which TPE is used beyond only renal indications, (2) restrict study design to controlled studies only (thereby excluding studies with a retrospective arm) to remove challenges of evaluating validity of results reported, and (3) limit the search to only two electronic databases to allow for manageable data analyses.

### 2.1 Study selection

We included all systematic reviews and controlled studies (randomized controlled trials [RCTs], controlled clinical trials, prospective comparative cohort studies) that assessed the use of a fluid intervention in any patient undergoing TPE, for any underlying medical condition. Although RCTs represent the ideal study design, additional study designs were included as not all clinical questions related to TPE can be answered with an RCT. We included studies that compared any fluid replacement to another fluid replacement therapy, placebo, or no fluid replacement (no intervention). Fluids included human albumin solution (HAS) only (any concentration), FFP only, other plasma derivatives, crystalloid only, colloid only, and a combination fluid.

We excluded studies where (a) TPE was used as treatment for TTP, as a relationship between outcomes and fluid type for this condition is well established,^9^ and (b) where hydroxyethyl starch (HES) was the sole replacement fluid as trial evidence suggests increased mortality and renal injury in ICU patients where it was used as a resuscitation fluid in severe sepsis.^12^ We recorded all outcomes reported by the included studies that relate to efficacy, safety, or patient‐reported measures. We also noted where a cost‐analysis had been undertaken.

### 2.2 Searches

On October 9, 2020 a single author (C.D.) searched MEDLINE (Ovid, 1946 onward) and Embase (Ovid, 1974 onward) for relevant studies (see Supporting Information for full search strategy). Non‐English language papers were not sought. The reference lists for all systematic reviews were hand searched for relevant studies by one author (R.K.).

### 2.3 Data collection and analysis

We used Covidence software for all screening and full text assessment. Two review authors (R.K. and L.J.G.) independently assessed all studies identified by the database searches, with conflict resolution by discussion. The same authors (R.K. and L.J.G.) independently extracted data using a standardized data extraction form (using Excel). Disagreements were resolved by discussion or through arbitration with the senior author (L.G.). The review authors were not blinded to names of authors, institutions, journals, or the study outcomes.

#### 2.3.1 Data synthesis

Data from RCTs, controlled clinical trials, and prospective studies were tabulated. Included studies were grouped according to the underlying medical condition for which TPE was indicated (eg, neurological, renal, hematological), and for each medical condition we categorized outcome measures into three major groups: (1) efficacy (clinical and laboratory outcomes), (2) safety (clinical and laboratory outcomes), and (3) patient‐reported outcomes. We also noted where a cost‐effectiveness or economic assessment had been performed. We examined the frequency and trends of outcomes reported: across all studies, and by the underlying condition. As this is a scoping review aiming to identify outcomes, results are presented narratively by type of outcome reported.

## 3 RESULTS

**FIGURE 1:**
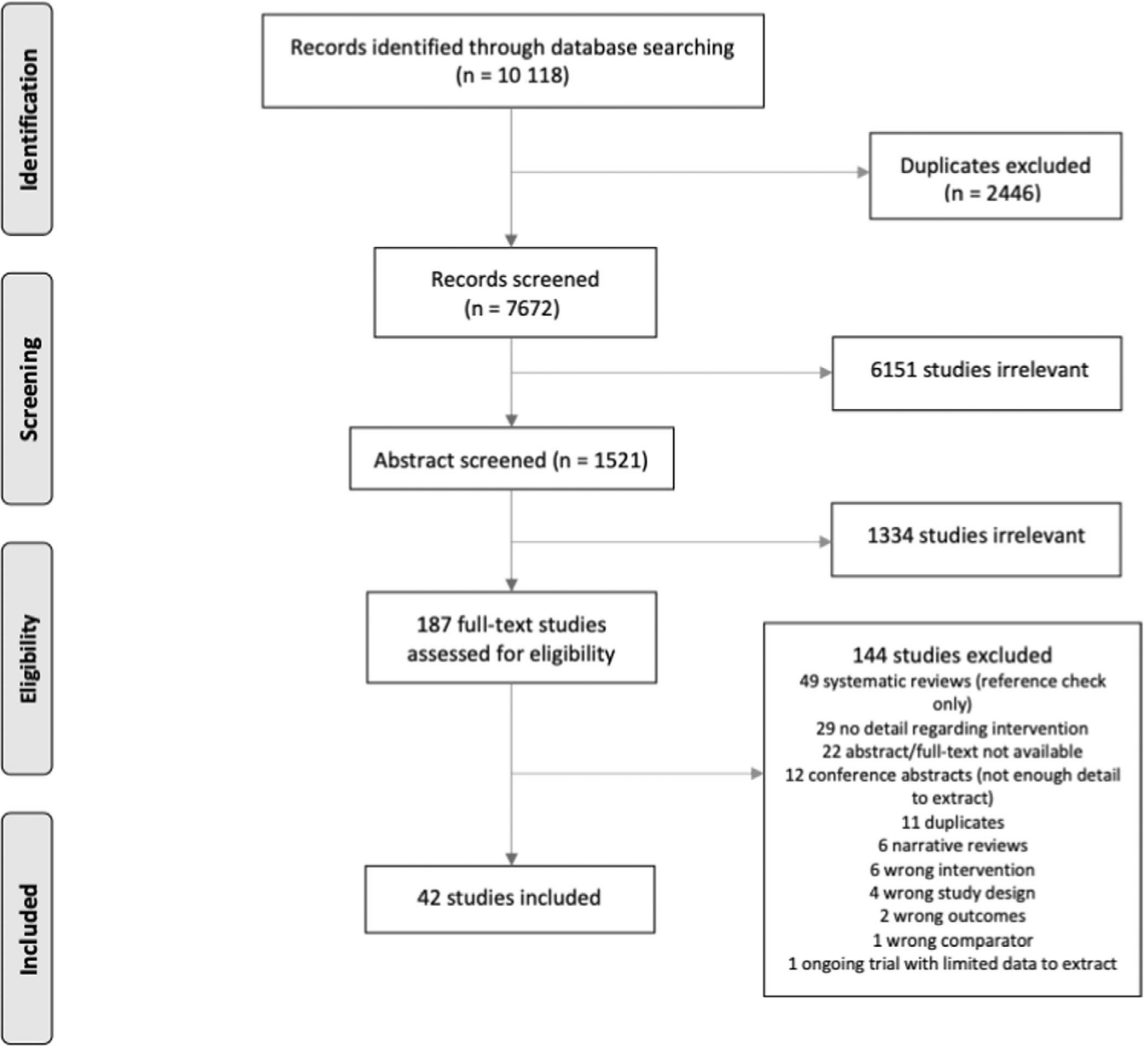
PRISMA flow diagram

After the removal of duplicates, we screened 7672 references based on title and abstract, and excluded 7485 references (Figure 1). We screened 187 titles at full text and excluded 144: 55 were systematic or narrative reviews, 22 had no abstract or full text available, 12 were only available as conference proceedings with limited detail. We included 42 studies of 1921 patients in the qualitative analysis (Figure 1).

### 3.1 Study characteristics

Forty‐two included studies fulfilled our predefined criteria. Thirty‐seven were RCTs,^13^, ^14^, ^15^, ^16^, ^17^, ^18^, ^19^, ^20^, ^21^, ^22^, ^23^, ^24^, ^25^, ^26^, ^27^, ^28^, ^29^, ^30^, ^31^, ^32^, ^33^, ^34^, ^35^, ^36^, ^37^, ^38^, ^39^, ^40^, ^41^, ^42^, ^43^, ^44^, ^45^, ^46^, ^47^, ^48^, ^49^ and five were prospective cohort studies.^7^, ^50^, ^51^, ^52^, ^53^ An overview of included studies can be seen in (Table S1).

Clinical conditions for the use of TPE included: neurological (18/42, 43%),^13^, ^14^, ^15^, ^16^, ^17^, ^18^, ^19^, ^20^, ^21^, ^22^, ^23^, ^24^, ^25^, ^26^, ^27^, ^28^, ^29^, ^30^ immunological (9/42, 21%),^31^, ^32^, ^33^, ^34^, ^35^, ^36^, ^37^, ^38^, ^39^ renal (4/42, 10%),^40^, ^41^, ^50^, ^51^ rheumatology (2/42, 5%),^42^, ^43^ hematological (2/42, 5%),^44^, ^52^ dermatological (1/42, 2%),^45^ and other categories (6/42, 14%).^7^, ^46^, ^47^, ^48^, ^49^, ^53^ The most common type of replacement fluids used were albumin (30/42, 71%),^7^, ^13^, ^14^, ^15^, ^16^, ^17^, ^19^, ^20^, ^24^, ^25^, ^26^, ^27^, ^29^, ^30^, ^31^, ^32^, ^33^, ^34^, ^35^, ^36^, ^37^, ^38^, ^41^, ^42^, ^45^, ^46^, ^47^, ^48^, ^50^, ^51^ albumin mix with other fluids (9/42, 21%),^7^, ^18^, ^21^, ^23^, ^39^, ^44^, ^46^, ^47^, ^48^ and FFP (4/42, 10%).^21^, ^46^, ^49^, ^53^ Six studies (14%)^7^, ^21^, ^38^, ^46^, ^47^, ^48^ used more than one replacement fluid.

Where stated, patients in 69% (29/42)^7^, ^13^, ^14^, ^15^, ^16^, ^18^, ^19^, ^21^, ^23^, ^24^, ^26^, ^27^, ^28^, ^30^, ^32^, ^35^, ^36^, ^37^, ^38^, ^39^, ^41^, ^42^, ^43^, ^44^, ^45^, ^46^, ^47^, ^48^, ^51^ of the studies were older than 16 years. Follow up duration varied in length with a median follow up of 6 months (IQR 1‐13). Duration of follow up was not reported in five studies^7^, ^40^, ^46^, ^50^, ^53^ (Table S1). One trial was assessed as ongoing, with no results currently available.^54^

### 3.2 Outcome(s)

#### 3.2.1 Primary outcomes reported

We assessed the primary outcomes across all conditions (see Table S1). Only eight studies (19%, 8/42)^13^, ^14^, ^18^, ^21^, ^24^, ^27^, ^30^, ^34^ defined primary outcomes and, where stated, were either categorized as efficacy (clinical) outcomes (five studies^18^, ^24^, ^27^, ^30^, ^34^), or patient reported outcomes (three studies).^13^, ^14^, ^21^ The majority of these studies were for a neurology indication (88% 7/8)^13^, ^14^, ^18^, ^21^, ^24^, ^27^, ^30^ with only one in immunology.^34^ A power calculation was reported in five of the neurology studies using various clinical and patient reported outcomes: expanded disability status scale^14^ and functional evaluation using visual analog scores^13^ (Multiple sclerosis), ability to walk^21^ (GBS), activities of daily living and cognitive scales (Alzheimer's),^24^ targeted neurological deficit^27^(chronic inflammatory polyneuropathy). The immunological study's power calculation was based on composite of death from any cause and end stage renal disease (ANCA‐vasculitis).^34^ The remaining two neurology studies defined their primary outcomes (expanded disability status scale, relapse rate, MRI images^18^; neuropathy disability^30^), but did not report a power calculation based on these (see Table S1).

##### Efficacy: Clinical

Only studies in neurology, rheumatology, hematology, and dermatology reported on disease‐specific clinical efficacy outcomes. No studies reported on non‐disease specific clinical efficacy outcomes. Studies in immunology and renal conditions did not report on any clinical efficacy outcomes (Table 1).

**TABLE 1:** Summary of efficacy, safety, and patient reported outcomes

Specialty (no. of studies)	Efficacy: disease specific (no. of studies)	Efficacy: non‐disease specific (no. of studies)	Safety: disease‐specific	Safety: treatment‐specific (no. of studies)	Patient‐reported outcomes (no. of studies)	Cost comparison (no. of studies)
Neurology (18)	*Clinical*: Neurological disability score (11) Neurological assessment (9) *Laboratory‐based*: CSF analysis (5) Nerve conduction studies (3) Brain MRI (2)	*Clinical*: No outcomes reported *Laboratory‐based*: Immunoglobulin levels (4) Complement levels (2) Lymphocyte subsets (1) Full blood count (1) Coagulation profile (1) Serum protein electrophoresis (1) Cryoglobulin levels (1) Plasminogen levels (1)	No outcomes reported	Hypotension (6) Allergic reaction (3) Infection (3) Bleeding (1) Febrile transfusion reaction (1)	Functional improvement score (7) Functional disability scale (2) Activity of daily living score (2)	Not reported
Immunology (9)	*Clinical*: No outcomes reported *Laboratory‐based*: dsDNA (2) ANCA (2) MRI (1)	*Clinical*: No outcomes reported *Laboratory‐based*: Immunoglobulin levels (5) C‐reactive protein (5) Serum creatinine (5) Complement levels (4) Coagulation profile (3) Plasma viscosity (2) Liver function tests (2) Haptoglobin (1) Urine protein (1) Serum electrolytes (1)	No outcomes reported	Allergic reaction (2) Hypotension (2) Febrile transfusion reaction (2) Infection (1)	Clinical symptom improvement (3) Quality of life score (1)	Not reported
Renal (4)	*Clinical*: No outcomes reported *Laboratory‐based*: Histological changes (2) Isoagglutinin titres (1)	*Clinical*: No outcomes reported *Laboratory‐based*: Renal function evaluation (2)	No outcomes reported	Bleeding (1)	No outcomes reported	Not reported
Rheumatology (2)	*Clinical*: Clinical evaluation (2) Joint scores (1) *Laboratory‐based*: Rheumatoid factor (1)	*Clinical*: No outcomes reported *Laboratory‐based*: Immunoglobulin levels (2) Complement levels (2) ESR (2) Coagulation profile (1) Liver function tests (1)	No outcomes reported	Febrile transfusion reaction (1)	Functional assessment score (1)	Not reported
Hematology (2)	*Clinical*: Requirement for dialysis (1) *Laboratory‐based*: Plasma viscosity (1)	*Clinical*: No outcomes reported *Laboratory‐based*: Hemoglobin (1)	No outcomes reported	No outcomes reported	No outcomes reported	Not reported
Dermatology (1)	*Clinical*: Visual analog scale (1) *Laboratory‐based*: No outcomes reported	*Clinical*: No outcomes reported *Laboratory‐based*: Full blood count (1) Coagulation profile (1) Serum protein electrophoresis (1)	No outcomes reported	Allergic reaction (1)	Not outcomes reported	Not reported
Other conditions (6)	*Clinical*: PELOD score (1) Fluid resuscitation volume (1) *Laboratory‐based*: No outcomes reported	*Clinical*: No outcomes reported *Laboratory‐based*: Oncotic pressure (3) Plasma viscosity (2) Full blood count (1) Serum creatinine (1) Arterial blood gas analysis (1) vWF levels (1) ADAMTS‐13 (1) Complement levels (1) Coagulation markers (1)	No outcomes reported	No outcomes reported	No outcomes reported	Cost analysis (1)

Abbreviations: ADAMTS‐13, A Disintegrin And Metalloproteinase with ThromboSpondin‐1 motifs; 13th member of the family; ANCA, anti‐neutrophil cytoplasm antibodies; CSF, cerebrospinal fluid; dsDNA, double stranded DNA; ESR: erythrocyte sedimentation rate; MRI, magnetic resonance imaging; PELOD, Paediatric Logistic Organ Dysfunction; vWF, von Willebrand factor antigen.

In patients with underlying neurology conditions, there was consensus on disease‐specific clinical outcome measures used, with neurological disability scores (11/18, 61%)^13^, ^14^, ^15^, ^16^, ^18^, ^19^, ^24^, ^25^, ^26^, ^27^, ^30^ and change in neurological assessment (9/18, 50%)^15^, ^19^, ^23^, ^24^, ^25^, ^26^, ^27^, ^28^, ^29^ being the most common outcomes measured. Clinical evaluation (ie, changes in symptoms and signs) was the single common disease‐specific outcome measure in rheumatology patients and was used in both RCTs included in this review.^42^, ^43^ There was no consensus on disease specific clinical efficacy outcome measures for patients with a hematological indication for TPE (Table 1).

##### Efficacy: Laboratory‐based

All studies except those in dermatology, and those classified under “other conditions” reported laboratory‐based disease‐specific efficacy outcomes.

Where reported, cerebrospinal fluid analysis (5/18, 28%)^15^, ^22^, ^24^, ^25^, ^26^ was the most common laboratory‐based disease‐specific outcome measure in patients undergoing plasma exchange for underlying neurological disease. In patients with immunological disease, laboratory‐based non‐disease‐specific outcome measures were frequently reported with changes in C‐Reactive Protein (CRP) (5/9, 56%)^32^, ^34^, ^37^, ^38^, ^39^ and immunoglobulin levels (5/9, 56%)^32^, ^35^, ^36^, ^38^, ^39^ used in the majority of RCTs. In patients with renal disease, laboratory‐based disease‐specific outcome measures were dependent on the specific underlying condition with evaluation of histological changes commonly used (2/4, 50%).^40^, ^41^ Changes in immunoglobulin, complement levels, and erythrocyte sedimentation rate (ESR) were used in all RCTs as non‐disease‐specific laboratory outcome measures in patients with rheumatoid arthritis.^42^, ^43^

##### Safety: Treatment‐specific

All studies, except in hematology and those classified under “other conditions” reported treatment‐specific safety outcomes.

The most common treatment‐specific (caused by the TPE itself or any part of the procedure) outcome reported was hypotension (neurology: six studies^14^, ^16^, ^17^, ^20^, ^21^, ^24^; immunology: two studies^38^, ^39^; hematology: one study^44^), allergic reaction (neurology: three studies^17^, ^21^, ^26^; immunology: two studies^38^, ^39^; dermatology: one study^45^), febrile transfusion reaction (neurology: one study^21^; immunology: two studies^38^, ^39^; rheumatology: one study^42^), and infection (neurology: three studies^14^, ^20^, ^21^; immunology: one study^36^). Hypotension was primarily reported in patients exchanged with albumin.^14^, ^16^, ^17^, ^20^, ^24^, ^38^, ^44^ Allergic reaction was regularly reported in patients undergoing exchange with plasma‐derived fluid and colloids, such as Gelofusine.^17^, ^21^, ^26^, ^38^, ^45^ (Tables 1 and S2–S8).

##### Safety: Disease‐specific

No studies reported on disease‐specific safety outcomes.

#### 3.2.2 Patient‐reported outcomes

Patient‐reported outcomes were reported for neurology (10 studies^16^, ^20^, ^21^, ^22^, ^23^, ^24^, ^25^, ^26^, ^27^, ^28^), immunology (three studies^32^, ^34^, ^38^), and rheumatology conditions (one study^42^). They were not reported in the renal, hematology, or dermatology studies. The most commonly assessed patient‐reported outcomes were functional disability/assessment scores (neurology: two studies,^24^, ^28^ rheumatology: one study^42^), functional/clinical improvement scores (neurology: seven studies,^20^, ^21^, ^22^, ^23^, ^24^, ^26^, ^28^ immunology: three studies^32^, ^34^, ^38^), and activities of daily living/quality of life scores (neurology: two studies^24^, ^25^; immunology: one study^34^) (see Tables 1 and S2–S8). Where a power calculation was performed, patient‐reported outcomes including functional improvement scores and disability scales were used in four of the five^13^, ^14^, ^21^, ^24^ neurology studies, highlighting the recognition of the importance of patient‐focused outcomes in this field, as well as the strength of data around validated scales to be able to use these in a trial power calculation.

#### 3.2.3 Cost‐effectiveness or cost comparison

One study of multiple underlying conditions examined cost‐effectiveness of the type of replacement fluid used^47^ (see Table 1).

### 3.3 Impact of fluid used

The replacement fluid type used was consistent with ASFA guidance^6^ and in the few studies, which did a head‐to‐head comparison of fluid types, none was found to be superior. Type of replacement fluid used was not found to influence which efficacy outcomes were measured. Safety outcome measures reported on were dependent on fluid type used for exchange, again an expected finding.

## 4 DISCUSSION

There is lack of evidence describing outcome measures of efficacy and safety with different replacement fluids used during TPE. In this first scoping review, we identified 42 studies that were eligible, and of these, neurological conditions were the most frequent circumstances for TPE (43%), followed by immunological (21%), and renal (10%) diseases. In 71% of the studies, albumin was the most widespread type of fluid used. However, very few studies (eight studies) had defined the primary outcomes and reported a power calculation (six studies), indicating significant limitation to answering the primary outcome of this review, inherent to the type of studies available in the literature.

In patients with underlying neurological conditions, there was more of a consensus on disease‐specific efficacy outcome measures: neurological disability scores (61%) and changes in neurological assessment (50%) were the most prevalent outcomes measured. It was in the same setting that patient‐reported outcomes (including functional improvement and functional disability scores) were also frequently reported, and most used when a power calculation was reported. However, validation and universal agreement of these scoring systems is needed to allow for comparison between studies, as different scoring systems used may not be comparable to each other.

In other clinical settings the disease‐specific outcomes for both efficacy, safety, and patient‐reported outcomes varied significantly, or were not reported at all. Further, there was no universal marker for laboratory‐based outcomes (both efficacy and safety) and establishing a standardized laboratory tests across different disease would be difficult as these outcomes are likely to be disease specific. Safety outcomes were mainly related to replacement fluid type rather than being disease‐specific, with hypotension being the most reported adverse event related to albumin, which was also the most common fluid type used. The use of laboratory markers to determine safety of a TPE fluid, could indeed be a viable option to use in a clinical trial for evaluation of fluid types, as they can be measured easily, repeatedly, and over a short period of time. However, future studies are needed to assess the association between these markers and efficacy/safety outcome measures in different settings.

There was insufficient reporting of cost analysis of different replacement fluids for different indications by studies included in this review, and therefore it is currently unknown if any of the regular fluid replacement options are more cost‐effective compared to another. In the UK albumin is the more costly (£67.50 for 500 mL^55^) than normal saline (£3.10 for 500 mL^56^). This is important to note as it may have implications on overall cost of intervention, as most patients need to undergo TPE multiple times due to the chronicity of their underlying disease. This also has implications for the design of future trials: the use of disease‐specific efficacy or safety outcomes will require longer follow up, which will translate into increased cost.

### 4.1 Strengths and limitations of this review

The review was performed in accordance with published guidelines for scoping reviews,^11^ using a systematic process of searching, screening, and data extraction. A comprehensive literature search with no participant age restrictions was performed. We limited the search to English language papers only, and unpublished or non‐indexed studies were not sought, so there may be a risk of missing data. We also limited the search criteria to only two electronic databases (to allow for manageable data analyses), however, reference lists of included studies and any relevant systematic reviews were hand searched for additional studies, so reducing the chance of missing vital evidence. Additionally, some of the indications only had single studies with small sample sizes (fewer than 100 patients) and therefore these data may not be truly representative of the entire population. Although median follow up duration for studies was 6 months, the interquartile range was wide (1‐13 months) reflecting differences in disease specific requirements, which could potentially limit establishment of a “consensus” in agreeing the timeline for measurement of follow‐up data.

### 4.2 Implications for future research

The evidence provided in this scoping review could be used to support the development of a core outcome set for use in TPE research. However, while more data are available in neurological conditions and patients with ABO‐incompatible renal transplantation, a consensus on outcome data for efficacy, safety, and patient‐related outcomes is lacking, and further research is needed to define these. It is likely that core outcome sets will need to be specific to each clinical indication and, as patient‐reported outcomes are an important measure patient engagement, will be instrumental in their development.

## 5 CONCLUSION

This scoping review demonstrates greater consensus amongst the included studies on disease‐specific clinical outcome measures for neurological conditions, with neurological disability scales and change in neurological examination being the most common outcomes measured that should be taken forward by neurology community for agreeing the core outcome set following TPE procedures for neurological conditions. Similarly in this review, the patient‐reported outcomes were better defined in patients with neurology conditions, but they were less well‐defined in other clinical conditions. Safety measures were poorly defined for all clinical settings, and studies that had reported these focused mainly on safety of the replacement fluid type than disease‐specific safety outcomes. Further, there was no universal marker for laboratory‐based outcomes for both efficacy and safety, apart from patients with renal disease undergoing ABO‐incompatible renal transplantation. In order to determine the efficacy and safety of different fluid type used for therapeutic plasma exchange, there is a need to reach a consensus on the outcome measures that should be measured in future studies, and the outcome of this review should provide the basis to develop the core outcome set for use in TPE research.

Potentially short‐term outcome measures could be used to determine safety and cost‐effectiveness of the intervention and may be the most feasible outcomes to use when deciding on trial outcomes allowing for shorter follow up, reducing overall cost.

## CONFLICT OF INTEREST

The authors declare no potential conflict of interest.

## Supporting information


**Data S1** Supporting Information

## Data Availability

The data that supports the findings of this study are available in the supplementary material of this article.
