# Supplementary material for: Assessing efficacy and safety of replacement fluids in therapeutic plasma exchange: A systematic scoping review of outcome measures used
Source: J Clin Apher. 2022 Jun 21;37(5):438–48. doi: 10.1002/jca.21996 (PMC9795884; doi:10.1002/jca.21996)
Supplement: Supplementary file 1 — Data S1 Supporting Information [file JCA-37-438-s001.docx]

Supplementary information

Contents

[Search strategies 2](#_Toc91692708)

[Data collection tool 5](#_Toc91692709)

[Table s1. Summary of included studies 6](#_Toc91692710)

[Table s2: Outcome measures: neurology 14](#_Toc91692711)

[Table s3: Outcome measures: immunology 16](#_Toc91692712)

[Table s4: Outcome measures: renal 18](#_Toc91692713)

[Table s5: Outcome measures: rheumatology 18](#_Toc91692714)

[Table s6: Outcome measures: haematology 20](#_Toc91692715)

[Table s7: Outcome measures: dermatology 20](#_Toc91692716)

[Table s8: Outcome measures: other conditions 22](#_Toc91692717)

## Search strategies

**MEDLINE (Ovid)**

1. Plasmapheresis/

2. Plasma Exchange/

3. (plasma exchange* or plasmapheres* or (plasma adj3 (pheres* or apheres* or replacement*)) or therapeutic apheres*).tw,kf.

4. 1 or 2 or 3

5. Systematic Review.pt.

6. Meta-Analysis.pt.

7. ((meta analy* or metaanaly*) and (trials or studies)).ab.

8. (meta analy* or metaanaly* or evidence-based).ti.

9. ((systematic* or evidence-based) adj2 (review* or overview*)).tw,kf.

10. (cochrane or medline or pubmed or embase or cinahl or cinhal or lilacs or "web of science" or science citation index or scopus or search terms or literature search or electronic search* or comprehensive search* or systematic search* or published articles or search strateg* or reference list* or bibliograph* or handsearch* or hand search* or manual* search*).ab.

11. Cochrane Database of systematic reviews.jn.

12. (additional adj (papers or articles or sources)).ab.

13. ((electronic* or online) adj (sources or resources or databases)).ab.

14. (relevant adj (journals or articles)).ab.

15. or/5-14

16. Review.pt.

17. RANDOMIZED CONTROLLED TRIALS AS TOPIC/

18. selection criteria.ab. or critical appraisal.ti.

19. (data adj (abstraction or extraction or analys*)).ab.

20. RANDOMIZED CONTROLLED TRIAL/

21. or/17-20

22. 16 and 21

23. 15 or 22

24. (Randomized Controlled Trial or Controlled Clinical Trial or Clinical Trial Protocol).pt.

25. (randomi* or randomly or placebo).tw,kf.

26. trial.ti,kf.

27. Clinical Trials as Topic/

28. Clinical Trial, Phase III/ or ("phase 3" or "phase3" or "phase III" or P3 or "PIII").tw,kf.

29. or/24-28

30. CONTROLLED BEFORE-AFTER STUDIES/

31. INTERRUPTED TIME SERIES ANALYSIS/

32. (nonrandom* or non random*).tw,kf.

33. (controlled clinical stud* or controlled stud* or controlled trial* or controlled clinical trial* or control group* or comparative stud* or comparative trial* or (compar* adj3 group*)).tw,kf.

34. (pre-post or pre-test* or pretest* or posttest* or post-test* or (pre adj5 post)).tw,kf.

35. ((before adj15 (after or during)) or "before-after" or time series or time point* or repeated measur*).tw,kf.

36. or/30-35

37. 23 or 29 or 36

38. (exp ANIMALS/ or exp ANIMAL EXPERIMENTATION/ or exp MODELS, ANIMAL/) not HUMANS/

39. Editorial.pt.

40. 38 or 39

41. 37 not 40

42. 4 and 41

43. limit 42 to english language

**Embase (Ovid)**

1. Plasmapheresis/

2. Plasma Exchange/

3. (plasma exchange* or plasmapheres* or (plasma adj3 (pheres* or apheres* or replacement*)) or therapeutic apheres*).tw,kw.

4. 1 or 2 or 3

5. Meta Analysis/

6. (meta analy* or metaanaly*).ti,kw.

7. ((meta analy* or metaanaly*) and (trials or studies)).ab.

8. Systematic Review/

9. ((systematic* or evidence-based) adj2 (review* or overview*)).tw,kw.

10. (evidence synthes* or cochrane or medline or pubmed or embase or cinahl or cinhal or lilacs or "web of science" or google scholar or google database or science citation index or scopus or search terms or literature search or electronic search* or comprehensive search* or systematic search* or published articles or search strateg* or reference list* or bibliograph* or handsearch* or hand search* or manual* search*).ab.

11. ((electronic* or online) adj (sources or resources or databases)).ab.

12. ((additional adj (papers or articles or sources)) or (relevant adj (journals or articles))).ab.

13. exp "Controlled Clinical Trial (Topic)"/

14. or/5-13

15. Review.pt.

16. (data extraction or selection criteria).ab.

17. 15 and 16

18. 14 or 17

19. Editorial.pt.

20. 18 not 19

21. (random* or factorial* or crossover* or cross over* or cross-over* or placebo* or doubl* blind* or singl* blind* or assign* or allocat* or volunteer*).mp.

22. randomized controlled trial/ or crossover-procedure/ or single-blind procedure/ or double-blind procedure/ or triple-blind procedure/

23. (nonrandom* or non random*).tw.

24. (controlled clinical stud* or controlled stud* or controlled trial* or controlled clinical trial* or control group* or comparative stud* or comparative trial* or (compar* adj3 group*)).tw,kw.

25. controlled clinical trial/

26. time series analysis/

27. pretest posttest control group design/ or pretest posttest design/

28. (pre-post or pre-test* or pretest* or posttest* or post-test* or (pre adj5 post)).tw.

29. ((before adj15 (after or during)) or "before-after" or time series or time point* or repeated measur*).tw.

30. 20 or 21 or 22 or 23 or 24 or 25 or 26 or 27 or 28 or 29

31. (exp animal/ or nonhuman/) not exp human/

32. Animal experiment/ not (human experiment/ or human/)

33. 31 or 32

34. 30 not 33

35. 4 and 34

36. limit 35 to english language

## Data collection tool

| Number of papers  Study type  Year |
| --- |
| Primary objective of the study |
| Study population |
| Fluid intervention type |
| Use of concomitant medication |
| Duration of intervention |
| Outcome measures  *I. Efficacy*  1. Disease specific  - clinical  - non-clinical  2. Non-disease specific  - clinical  - non-clinical  *II. Safety*  Adverse outcomes   - Treatment specific (e.g., bleeding, allergy, hypotension) - Disease specific   *III. Cost-related* |
| Key findings |

## Table s1. Summary of included studies

| Author | Year | Country | Study Type | Indication (ASFA category^) | Sample size | Age | Replacement fluid and number of treatment sessions^ | Primary outcome (and power calculation, if reported) | Duration of follow-up |
| --- | --- | --- | --- | --- | --- | --- | --- | --- | --- |
| NEUROLOGY | | | | | | | | | |
| Canadian cooperative trial | **1985** | Canada | RCT  Multi-centre | Multiple sclerosis  (ASFA II/III) | 57 | >16 years | Albumin  12 sessions | Treatment failure by neurological disability scale (EDSS)  Power calculation based on detection of 30% difference in treatment failure between groups | >12 months |
| Brochet | **2020** | France | RCT  Multi-centre | Multiple sclerosis  (ASFA II/III) | 14 | >16 years | Albumin  6 sessions | Moderate improvement in functional evaluation (VAS)  Power calculation based on at least 30% improvement in treatment group | 6 months |
| Gordon | **1985** | Canada | RCT  Single centre | Multiple sclerosis  (ASFA II/III) | 10 | 23-47 years | Albumin  8 sessions | Not defined | 6 months |
| Khatri | **1984** | USA | RCT  Single centre | Multiple sclerosis  (ASFA II/III) | 26 | >16 years | Albumin  20 sessions | Not defined | 11 months |
| Noseworthy | **1989** | Canada | RCT  Multi-centre | Multiple sclerosis  (ASFA II/III) | 25 | Not stated | Albumin  20 sessions | Not defined | 24 months |
| Tabrizi | **2010** | Iran | RCT  Single centre | Multiple sclerosis  (ASFA II/III) | 18 | >16 years | Albumin mix  5 sessions | Changes in EDSS score, relapse rate and MRI images  No power calculation | 9 months |
| Weiner | **1989** | USA | RCT  Multi-centre | Multiple sclerosis  (ASFA II/III) | 59 | 18-50 years | Albumin  11 sessions | Not defined | 24 months |
| North American study group | **1985** | USA  Canada | RCT  Multi-centre | Guillain Barre Syndrome  (ASFA I) | 122 | All ages | Albumin  3-5 sessions | Not defined | 6 months |
| French cooperative group | **1981** | France  Switzerland | RCT  Multi-centre | Guillain Barre Syndrome  (ASFA I) | 109 | >16 years | Albumin mix  FFP  4 sessions | Recover ability to walk.  Power calculation based on severity (mild, moderate, severe) of inability to walk | >12 months |
| McKhann | **1987** | USA | RCT  Single centre | Guillain Barre Syndrome  (ASFA I) | 122 | Not stated | Plasma derivative | Not defined | 6 months |
| Osterman | **1984** | Sweden | RCT  Multi-centre | Guillain Barre Syndrome  (ASFA I) | 18 | 16-77 years | Albumin mix  >5 sessions | Not defined | 2 months |
| Boada | **2020** | Spain  USA | RCT  Multi-centre | Alzheimer’s Disease | 347 | 55-85 years | Albumin  18 sessions | Change from baseline in activities of daily living scores (ADCS-ADL) and functional improvement scores (ADAS-Cog)  Power calculation based on detecting a difference in the change from baseline of 3 points on the ADAS-Cog, between the treatment groups | 14 months |
| Lopez  *(Abstract only)* | **2015** | Spain | RCT  Single centre | Alzheimer’s Disease | 18 | All ages | Albumin  14 sessions | Not defined | 6 months |
| Hahn | **1994** | Canada | RCT  Multi-centre | Chronic Inflammatory polyneuropathies  (ASFA I) | 18 | >18 years | Albumin  10 sessions | Not defined | 15-56 months |
| Weinshenker | **1999** | USA | RCT  Single centre | Chronic Inflammatory polyneuropathies  (ASFA I) | 22 | >16 years | Albumin  7 sessions | Targeted neurological deficit  Power calculation based on a success rate of 70% in treatment group versus 20% in sham exchange group | 7 months |
| Greenwood | **1985** | UK | RCT  Multi-centre | Chronic Inflammatory polyneuropathies  (ASFA I) | 14 | >16 years | Colloid mix  5 sessions | Not defined | 12 months |
| Dyck | **1991** | USA | RCT  Single centre | Polyneuropathy associated with paraproteinemia  (ASFA I) | 19 | Not stated | Albumin  6 sessions | Not defined | 3 weeks |
| Oksenhendler | **1995** | France | RCT  Multi-centre | Polyneuropathy associated with paraproteinemia  (ASFA I) | 22 | >16 years | Albumin  15 sessions | Neuropathy disability score after 12 months of treatment  No power calculation | 12 months |
| IMMUNOLOGY | | | | | | | | | |
| Tsokos | **1981** | USA | RCT  Single centre | Systemic Lupus Erythematosus  (ASFA II) | 9 | Not stated | Albumin | Not defined | 2 weeks |
| Wei | **1983** | USA | RCT  Single centre | Systemic Lupus Erythematosus  (ASFA II) | 18 | 18-60 years | Albumin | Not defined | 1 month |
| Pohl | **1991** | USA | RCT  Multi-centre | Systemic Lupus Erythematosus  (ASFA II) | 40 | Not stated | Albumin | Not defined | > 12 months |
| Walsh | **2020** | Canada  Australia  Belgium  Czech Republic  Europe  Japan  UK  USA | RCT  Multi-centre | Anti-neutrophil cytoplasm antibodies vasculitis  (ASFA I/III) | 352 | >15 years | Albumin | Composite of death from any cause or ESRD  Power calculation was based on this outcome | 7 years |
| McCune | **1982** | USA | RCT  Single centre | Raynauds  (ASFA III) | 6 | >16 years | Albumin | Not defined | 12 months |
| Miller | **1992** | USA | RCT  Single centre | Polymyositis/ dermatomyositis | 9 | >16 years | Albumin | Not defined | 1 month |
| Szpirt | **2011** | Denmark | RCT  Single centre | Wegeners  (ASFA I/III) | 16 | >16 years | Albumin | Not defined | 5 years |
| Bambauer | **1999** | Germany | RCT  Single centre | Multiple causes | 24 | 16-65 years | Albumin  Protein solution  15 sessions | Not defined | 8 weeks |
| Keller | **2000** | Germany | RCT  Single centre | Multiple causes | 24 | >16 years | Albumin mix  3 sessions | Not defined | 6-9 days |
| RENAL | | | | | | | | | |
| Hanaoka | **2019** | Japan | Prospective cohort  Single centre | ABOi renal transplant  (ASFA I) | 29 | Not stated | Albumin  14 sessions | Not defined | Not stated |
| Yamada | **2015** | USA | Prospective cohort  Single centre | ABOi renal transplant  (ASFA I) | 9 | >18 years | Albumin | Not defined | 48 hours |
| Kirubakaran | **1981** | Australia | RCT  Single centre | Antibody mediated renal allograft rejection  (ASFA II) | 11 | Not stated | Protein solution | Not defined | Not stated |
| Pusey | **1991** | UK | RCT  Single centre | FSGN  (ASFA I/II/III) | 25 | 16-65 years | Albumin | Not defined | 5 years |
| RHEUMATOLOGY | | | | | | | | | |
| Dwosh | **1983** | Canada | RCT  Single centre | Rheumatoid arthritis  (ASFA IV) | 20 | 16-65 years | Albumin  10 sessions | Not defined | 4 months |
| Rothwell | **1979** | Canada | RCT  Single centre | Rheumatoid arthritis  (ASFA IV) | 10 | >16 years | Crystalloid mix  6 sessions | Not defined | 21 days |
| HAEMATOLOGY | | | | | | | | | |
| Bada | **1992** | USA | Prospective cohort  Single centre | Polycythaemic viscosity syndrome  (ASFA I) | 71 | Infants | Plasma derivative | Not defined | 5 years |
| Zucchelli | **1984** | Italy | RCT  Single centre | Myeloma with cast nephropathy  (ASFA II) | 15 | 48-73 years | Albumin mix  5 sessions | Not defined | 3-6 years |
| DERMATOLOGY | | | | | | | | | |
| Clemmensen | **1983** | Denmark | RCT  Single centre | Psoriasis  (ASFA IV) | 3 | 25-69 years | Albumin  7 sessions | Not defined | 7 weeks |
| OTHERS | | | | | | | | | |
| Arslan | **2004** | Turkey | RCT  Single centre | Multiple indications* | 23 | 16-65 years | Albumin  Albumin mix  FFP  2 sessions | Not defined | Not stated |
| LeConte | **1997** | France | RCT  Single centre | Multiple indications* | 27 | >16 years | Albumin  Albumin mix | Not defined | 24 hours |
| Tek | **2004** | Turkey | Prospective cohort  Single centre | Multiple indications* | 42 | 16-76 years | Albumin  Albumin mix  2 sessions | Not defined | Not stated |
| Tek | **2003** | Turkey | RCT  Single centre | Multiple indications* | 29 | 25-73 years | Albumin  Albumin mix  2 sessions | Not defined | 24 hours |
| Fortenberry | **2019** | USA | Prospective cohort  Single centre | Thrombocytopenia associated with multi-organ failure | 60 | 1 week – 21 years | FFP  5 sessions | Not defined | Not stated |
| Kravitz | **1989** | USA | RCT  Single centre | Burn shock  (ASFA III) | 9 | 15-60 years | FFP | Not defined | 48 hours |

RCT, randomised controlled trial; FFP, fresh frozen plasma; ADCS-ADL, Alzheimer’s Disease Cooperative Study – Activities of Daily Living Scale; ADAS-Cog, Alzheimer’s Disease Assessment Scale – Cognitive Subscale; EDSS, Expanded Disability Status Scale; VAS, visual analogue scale; ESRD, end stage renal disease; ASFA, American Society for Apheresis; ^ where stated

ASFA categories: I, apheresis accepted as first line therapy (primary or in combination with other treatments); II, second-line therapy (primary or in combination with other treatments); III, optimum role of apheresis not established; IV, apheresis ineffective or harmful^1^

* auto- immune haemolytic anaemia, familial hypercholesterolemia, hyperbilirubinemia, hypoxic encephalopathy, disseminated intravascular coagulopathy, sepsis, acute myeloid leukaemia, chronic myeloid leukaemia, tetanus, HELLP syndrome, hepatic encephalopathy, systemic lupus erythematosus, myasthenia gravis, recurrent iritis, uveitis, Wegener’s granulomatosis, haemolytic uremic syndrome after bone marrow transplantation, graft versus host disease, suspicion of Felty syndrome, cryoglobulinemia, pemphigus vulgaris, nephrotic syndrome with membranous glomerulonephritis.

## Table s2: Outcome measures: neurology

| Condition | Study design (no. of studies) | Efficacy: Disease specific, Clinical  (no. of studies) | | | Efficacy: Disease specific, Laboratory-based  (no. of studies) | Efficacy: Non-disease specific, Clinical  (no. of studies) | Efficacy: Non-disease specific, Laboratory-based  (no. of studies) | Safety: treatment specific  (no. of studies) | Safety: disease specific  (no. of studies) | Patient-reported outcome  (no. of studies) |
| --- | --- | --- | --- | --- | --- | --- | --- | --- | --- | --- |
| Multiple Sclerosis (MS) | RCT (7)  N=209 | Neurology disability score (6)  Neurological assessment (2) | Brain MRI (1)  CSF analysis (2) | | | NR (not reported) | Immunoglobulin levels (2)  Complement levels (2)  Lymphocyte subsets (1) | Bleeding (1)  Infection (1)  Allergic reaction (1)  Hypotension (3) | NR (not reported) | Functional disability scale (2)  Activity of daily living scale (1) |
| Guillain Barre Syndrome (GBS) | RCT (4)  N=371 | Neurological assessment (1) | | CSF analysis (1) | | NR (not reported) | NR (not reported) | Infection (2)  Allergic reaction (1)  Hypotension (2)  Febrile transfusion reaction (1) | NR (not reported) | Functional improvement score (4) |
| Chronic Inflammatory polyneuropathies | RCT (3)  N=54 | Neurology disability score (2)  Neurological assessment (3)  Nerve conduction studies (1) | | NR (not reported) | | NR (not reported) | Immunoglobulin levels (1) | Anaphylaxis (1) | NR (not reported) | Functional improvement score (2)  Activity limitation score (1) |
| Alzheimer’s Disease (AD) | RCT (2)  N=365 | Neurology disability score (2)  Neurological assessment (2) | | Use of brain imaging, MRI (1)  CSF Tau A-beta proteins (2) | | NR (not reported) | Full blood count (1)  Coagulation factors (PT, aPTT, fibrinogen) (1) | Hypotension (1) | NR (not reported) | Functional improvement score (1)  Quality-of-life scores (2) |
| Polyneuropathy associated with paraproteinemia | RCT (2)  N=41 | Nerve conduction studies (2)  Neuropathy disability score (1)  Neurological assessment (1) | | Anti-myelin antibodies (1) | | NR (not reported) | Plasminogen levels (1)  Serum electrophoresis (1)  Cryoglobulin levels (1)  Immunoglobulin levels (1) | NR (not reported) | NR (not reported) | NR (not reported) |

N: total number of patients; RCT, randomised controlled trial; CSF, cerebrospinal fluid; MRI, magnetic resonance imaging; PT, prothrombin time; aPTT, activated partial thromboplastin time

## Table s3: Outcome measures: immunology

|  | Study design (no. of studies) | Efficacy: Disease specific, clinical  (no. of studies) | Efficacy: Disease specific, laboratory based  (no. of studies) | Efficacy: Non-disease specific, clinical  (no. of studies) | Efficacy: Non-disease specific, laboratory based  (no. of studies) | Safety: treatment specific  (no. of studies) | Safety: disease specific  (no. of studies) | Patient-reported outcome  (no. of studies) |
| --- | --- | --- | --- | --- | --- | --- | --- | --- |
| Systemic Lupus Erythematosus (SLE) | RCT (3)  N=67 | NR (not reported) | dsDNA titre (1) | NR (not reported) | Immunoglobulin levels (2)  Complement levels (2)  CRP (1)  Coagulation factors (PTT, fibrinogen) (1)  Markers of haemolysis (LDH, haptoglobin) (1)  Liver function tests (1)  Serum creatinine (2) | Infection (1) | NR (not reported) | Clinical symptom improvement (1) |
| ANCA vasculitis | RCT (1)  N=352 | NR (not reported) | ANCA titre | NR (not reported) | Serum creatinine  CRP/ESR  Haemoglobin | NR (not reported) | NR (not reported) | Quality of life score  Clinical symptom improvement |
| Raynaud’s | RCT (1)  N=6 | NR (not reported) | NR (not reported) | NR (not reported) | Immunoglobulin levels  Plasma viscosity | NR (not reported) | NR (not reported) | NR (not reported) |
| Polymyositis/ dermatomyositis | RCT (1)  N=9 | NR (not reported) | MRI  ANCA titres | NR (not reported) | Serum creatinine  CRP  Urine protein | NR (not reported) | NR (not reported) | NR (not reported) |
| Wegener’s | RCT (1)  N=16 | NR (not reported) | NR (not reported) | NR (not reported) | Immunoglobulin levels  Plasma viscosity | NR (not reported) | NR (not reported) | NR (not reported) |
| Multiple causes^ | RCT (2)  N=48 | NR (not reported) | dsDNA (1) | NR (not reported) | Immunoglobulin levels (2)  Complement levels (2)  CRP (2)  Coagulation factors (aPTT, fibrinogen) (2)  Liver function tests (1)  Serum creatinine (1)  Electrolytes (Na, K) (1) | Allergic reaction (2)  Hypotension (2)  Febrile transfusion reaction (2) | NR (not reported) | Clinical symptom improvement (1) |

ANCA, anti-neutrophil cytoplasm antibodies; N: total number of patients; RCT, randomised controlled trial; CRP, C-reactive protein; LDH, lactate dehydrogenase; dsDNA, double stranded DNA; Na, sodium, K, potassium; aPTT, activated partial thromboplastin time; ESR: Erythrocyte sedimentation rate

^ systemic lupus erythematosus, myasthenia gravis, recurrent iritis, uveitis, Wegener’s granulomatosis, haemolytic uremic syndrome (HUS) after bone marrow transplantation (BMT), graft versus host disease (GVHD), suspicion of Felty syndrome, cryoglobulinemia, pemphigus vulgaris, acute myeloid leukaemia (AML), nephrotic syndrome with membranous glomerulonephritis (GN), chronic myeloid leukaemia (CML)

## Table s4: Outcome measures: renal

|  | Study design (no. of studies) | Efficacy: Disease specific, clinical  (no. of studies) | Efficacy: Disease specific, laboratory based  (no. of studies) | Efficacy: Non-disease specific, clinical  (no. of studies) | Efficacy: Non-disease specific, laboratory based  (no. of studies) | Safety: treatment specific  (no. of studies) | Safety: disease specific  (no. of studies) | Patient-reported outcome  (no. of studies) |
| --- | --- | --- | --- | --- | --- | --- | --- | --- |
| ABOi renal transplant | Prospective cohort ( 2)  N=38 | NR (not reported) | Isoagglutinin titres (1) | NR (not reported) | NR (not reported) | Bleeding (1) | NR (not reported) | NR (not reported) |
| Antibody mediated renal allograft rejection | RCT (1)  N=11 | NR (not reported) | Histological changes | NR (not reported) | Serum urea | NR (not reported) | NR (not reported) | NR (not reported) |
| Focal necrotising glomerulonephritis (FSGN) | RCT (1)  N=25 | NR (not reported) | Histological changes | NR (not reported) | Serum creatinine  Creatinine clearance | NR (not reported) | NR (not reported) | NR (not reported) |

N: total number of patients; RCT, randomised controlled trial

## Table s5: Outcome measures: rheumatology

|  | Study design (no. of studies) | Efficacy: Disease specific, clinical  (no. of studies) | Efficacy: Disease specific, laboratory based  (no. of studies) | Efficacy: Non-disease specific, clinical  (no. of studies) | Efficacy: Non-disease specific, laboratory based  (no. of studies) | Safety: treatment specific  (no. of studies) | Safety: disease specific  (no. of studies) | Patient-reported outcome  (no. of studies) |
| --- | --- | --- | --- | --- | --- | --- | --- | --- |
| Rheumatoid arthritis | RCT (2)  N=30 | Clinical evaluation (2)  Joint scores (1) | Rheumatoid factor (1) | NR (not reported) | Immunoglobulin levels (2)  Complement levels (2)  ESR (2)  Coagulation factors (PT, aPTT) (1)  Liver function tests (1) | Febrile transfusion reaction (1) | NR (not reported) | Functional assessment score (1) |

N: total number of patients; RCT, randomised controlled trial; PT, prothrombin time; aPTT, activated partial thromboplastin time; ESR: Erythrocyte sedimentation rate

## Table s6: Outcome measures: haematology

|  | Study design (no. of studies) | Efficacy: Disease specific, clinical  (no. of studies) | Efficacy: Disease specific, laboratory based  (no. of studies) | Efficacy: Non-disease specific, clinical  (no. of studies) | Efficacy: Non-disease specific, laboratory based  (no. of studies) | Safety: treatment specific  (no. of studies) | Safety: disease specific  (no. of studies) | Patient-reported outcome  (no. of studies) |
| --- | --- | --- | --- | --- | --- | --- | --- | --- |
| Polycythaemic viscosity syndrome | Prospective cohort (1)  N=71 | NR (not reported) | Plasma viscosity | NR (not reported) | Haemoglobin | NR (not reported) | NR (not reported) | NR (not reported) |
| Myeloma with cast nephropathy | RCT (1)  N=15 | Requirement for dialysis | Beta-2-microglobulin | NR (not reported) | NR (not reported) | NR (not reported) | NR (not reported) | NR (not reported) |

N: total number of patients; RCT, randomised controlled trial

## Table s7: Outcome measures: dermatology

|  | Study design (no. of studies) | Efficacy: Disease specific, clinical  (no. of studies) | Efficacy: Disease specific, laboratory based  (no. of studies) | Efficacy: Non-disease specific, clinical  (no. of studies) | Efficacy: Non-disease specific, laboratory based  (no. of studies) | Safety: treatment specific  (no. of studies) | Safety: disease specific  (no. of studies) | Patient-reported outcome  (no. of studies) |
| --- | --- | --- | --- | --- | --- | --- | --- | --- |
| Psoriasis | RCT (1)  N=3 | Visual analogue scale | NR (not reported) | NR (not reported) | Full blood count  Coagulation factors (aPTT, AT)  Serum protein electrophoresis | Allergic reaction | NR (not reported) | NR (not reported) |

N: total number of patients; RCT, randomised controlled trial; aPTT, activated partial thromboplastin time; AT, antithrombin

## Table s8: Outcome measures: other conditions

|  | Study design (no. of studies) | Efficacy: Disease specific, clinical  (no. of studies) | Efficacy: Disease specific, laboratory based  (no. of studies) | Efficacy: Non-disease specific, clinical  (no. of studies) | Efficacy: Non-disease specific, laboratory based  (no. of studies) | Safety – treatment specific  (no. of studies) | Safety – disease specific  (no. of studies) | Patient reported outcome  (no. of studies) |
| --- | --- | --- | --- | --- | --- | --- | --- | --- |
| Thrombocytopenia associated with multi-organ failure | Prospective cohort (1)  N=60 | PELOD score | NR (not reported) | NR (not reported) | Full blood count  Serum creatinine  Arterial blood gas analysis | NR (not reported) | NR (not reported) | NR (not reported) |
| Burn shock | RCT (1)  N=9 | Fluid resuscitation volume | NR (not reported) | NR (not reported) | vWF  ADAMTS-13 | NR (not reported) | NR (not reported) | NR (not reported) |
| Multiple indications^ | RCT (3)  Prospective cohort (1)  N=121 | NR (not reported) | NR (not reported) | NR (not reported) | Complement levels (1)  Coagulation markers (1)  Oncotic pressure (3)  Plasma viscosity (2) | NR (not reported) | NR (not reported) | NR (not reported) |

N: total number of patients; RCT, randomised controlled trial; vWF, von Willebrand factor antigen; ADAMTS-13, A Disintegrin And Metalloproteinase with ThromboSpondin‐1 motifs; 13th member of the family; PELOD, Paediatric Logistic Organ Dysfunction

^ auto- immune haemolytic anaemia, familial hypercholesterolemia, hyperbilirubinemia, hypoxic encephalopathy, disseminated intravascular coagulopathy, sepsis, AML, CML, tetanus, HELLP syndrome, hepatic encephalopathy

**References**

1. Padmanabhan A, Connelly-Smith L, Aqui N, et al. Guidelines on the Use of Therapeutic Apheresis in Clinical Practice - Evidence-Based Approach from the Writing Committee of the American Society for Apheresis: The Eighth Special Issue. *Journal of Clinical Apheresis* 2019;34(3):171-354. doi: <https://dx.doi.org/10.1002/jca.21705>
